# The research on gene-disease association based on text-mining of PubMed

**DOI:** 10.1186/s12859-018-2048-y

**Published:** 2018-02-07

**Authors:** Jie Zhou, Bo-quan Fu

**Affiliations:** Guangdong Key Laboratory of Computer Network, School of Computer Science and Engineering, South China University of Technology, Guangzhou, 510006 China

**Keywords:** MeSH, TF-IDF, Text mining, Human disease

## Abstract

**Background:**

The associations between genes and diseases are of critical significance in aspects of prevention, diagnosis and treatment. Although gene-disease relationships have been investigated extensively, much of the underpinnings of these associations are yet to be elucidated.

**Methods:**

A novel method integrates MeSH database, term weight (TW), and co-occurrence methods to predict gene-disease associations based on the cosine similarity between gene vectors and disease vectors. Vectors are transformed from the texts of documents in the PubMed database according to the appearance and location of the gene or disease terms. The disease related text data has been optimized during the process of constructing vectors.

**Results:**

The overall distribution of cosine similarity value was investigated. By using the gene-disease association data in OMIM database as golden standard, the performance of cosine similarity in predicting gene-disease linkage was evaluated. The effects of applying weight matrix, penalty weights for keywords (PWK), and normalization were also investigated. Finally, we demonstrated that our method outperforms heterogeneous network edge prediction (HNEP) in aspects of precision rate and recall rate.

**Conclusions:**

Our method proposed in this paper is easy to be conducted and the results can be integrated with other models to improve the overall performance of gene-disease association predictions.

## Background

In the medical research, an understanding of the association between genes and diseases is a crucial step toward prevention, diagnosis, and therapy of diseases. Although such gene-disease relationships have been investigated in many studies, the complex mechanism from genotype to phenotype and details of the genetic basis for diseases are still unrevealed. Furthermore, identifying all possible relationships by wet experimental methods are currently too expensive and time-consuming to be a feasible approach in consideration. To fill this gap, the bioinformatics-based approach may provide some candidate gene-disease linkages before employing large-scale population based epidemiological analysis.

In the recent decades, data-mining approaches, include the graph, machine learning, and text mining methods, had been proposed to study the gene-disease association [1–8]. Based on graph theory, the graph method constructs graphical models and several algorithms have been proposed like neighbor association [1], shortest path [2, 3], walking model [4], random surfer model [5], and network propagation model [6]. However, the power of the graph method may be limited in investigating less-studied genes or diseases [7, 8]. The machine learning method (MLM) explores associations between characteristic vectors reduced from genes and diseases. However, due to the specificity and structure of the data format used in MLM, a high quality data is required. In addition, to our knowledge, there is no best method for formatting or quantifying data, especially, disease data. As a consequence, the general application of MLM in deciphering gene-disease associations may be limited due to the availability of source data.

Text mining method had been applied in studying various biological problems like functional genomics [9], biological pathways [10], protein-protein interactions [11], protein representation [12], drug-gene association [13], comparative toxicogenomics [14, 15], neuropsychiatric disorder [16], and other areas in the biomedical domain [17] including large-scale bioinformatics analyses [8, 18–32]. DISEASES predicted the association through the co-occurrence method [21]. MimMiner [28] transformed OMIM [29] text to a relationship matrix and quantified the association among diseases using the term frequency–inverse document frequency method (TF-IDF). CATAPULT [8] and Heterogeneous Network Edge Prediction (HNEP) [30] integrated the graphic model and machine learning method, IMC [31] used a semi-supervised machine learning method, and LGscore [32] associated genes with disease through a Google search engine to predict associations between genes and diseases.

**Fig. 1:**
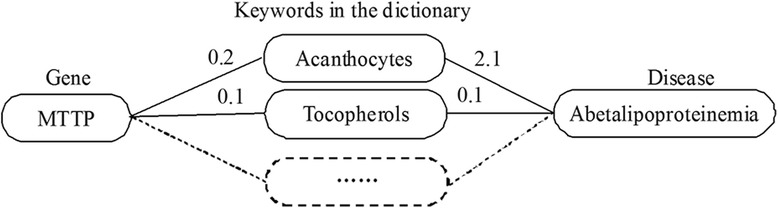
Use of keywords in the dictionary to describe genes and diseases

**Fig. 2:**
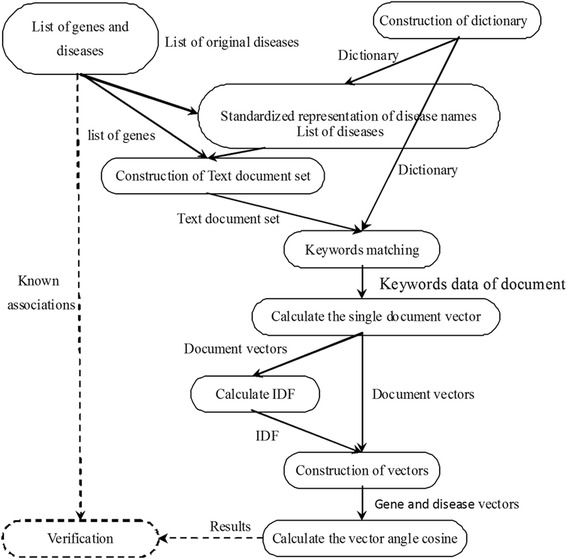
Flow chart representing the data processing steps

However, these methods did not integrate other valuable information that can be curated from other databases, such as MeSH, to improve accuracy or efficiency [27]. Moreover, the gene-disease co-occurrence ratio is usually low and this leads to a huge amount of text document sets needed to be curated to achieve the effective sample size. Therefore, in this study, we demonstrate an efficient data mining approach of deciphering gene-disease association by integrating the MeSH database and TF-IDF methods (Fig. 1). We transformed keywords in the dictionary to describe each of 3288 genes and 445 diseases, respectively, in a vector form and measured associations between genes and diseases using cosine similarity. The prediction performance was evaluated based on the accuracy and recall. Finally, our method was compared with HNEP [30] (Fig. 2).

## Methods

### Public data sources

The gene-disease linkages, including genes’ ID and disease names were curated from OMIM. Among all genes and diseases from OMIM, a total of 3288 genes and 445 diseases were also found in MeSH and used for analysis. The dictionary and the text document set were constructed according to MeSH and the content of abstract in PubMed, respectively. Although there were 16 categories at the first level of MeSH, we only used 5 categories, anatomy, organisms, diseases, chemicals and drugs, and psychiatry and psychology, of gene-disease associations relevant to construct the vector. Text files which not related with genes or diseases were removed. In total, the dictionary contained 27,453 keywords mapping to 56,341 nodes in MeSH. The text document set contained 528,878 associated with 3288 genes and 1,435,091 text files associated with 445 diseases, respectively.

### Data preprocessing

The relationship between N keywords was represented as the matrix form in N x N dimension and each element represented the association strength between keywords. The detailed steps are depicted schematically in Fig. 2.

### Text file vector construction

Each text file was transformed into three vectors, the vector of title, the vector of sentences in the abstract, and the vector of MeSH terms, respectively. The vectors of title represented the frequency of keywords occurred in the title. The vectors of sentences in the abstract represented sentences in the abstract. The vector of MeSH terms was coded binary: 1, if the keyword occurred, and 0, if not. Three vectors were then combined into one representative vector of the text file by the co-occurrence method (Table 1). We assigned a higher weight value for MeSH terms because these data had already been carefully annotated with respect to gene-disease relationships. Similarly, the gene-disease association based on their co-occurrence in the title would be stronger than the association based on sentences in the abstract. To reduce the bias article length, we normalized the representative vector by scaling the sum of all values of the text vector to 1.

**Table 1:** Weight values for the vector combination in this study

Vectors	Weight	Weight of abstract vectors with corresponding gene/disease in sentence	Weight of abstract vectors without corresponding gene/disease in sentence	Weight of MeSH terms vectors
In MeSH terms	3	3	2	3
In title	2	2	1	3
In abstract	1	2	1	2

### Term weight (TW) of keyword

We calculated the inverse document frequency (IDF) of keyword (eq. 1)

1 $$ {IDF}_i=\sqrt{\frac{1}{\sum {w}_i}} $$

in which *i* represents keyword and ∑*w*_*i*_ represents the sum of weighted values.

IDF was used to represent the importance of a keyword in aspects of gene or disease. If a keyword occurred more frequently among vectors, the IDF of this keyword would be smaller. We calculate penalty weights for keywords, PWK_*i*_, to weight the distance of a keyword to the MeSH root as eq. 2:

2 $$ {PWK}_i=\left\{\begin{array}{c}{2}^{T_i-5}\\ {}1\end{array}\right.\kern0.5em {\displaystyle \begin{array}{c}\left({T}_i<5\right)\\ {}\left({T}_i>=5\right)\end{array}} $$

where *T*_*i*_ represents the depth of the keyword in the MeSH tree.

If a keyword occurred at 5th or higher levels, no penalty it was applied. Otherwise, the weight would decrease to half in each level. The final weight value of the keyword was calculated as the product of IDF and PWK (eq. 3):

3 $$ {TW}_i={IDF}_i\cdot {PWK}_i $$

### Constructions of gene and disease vectors and correlation measurement

We transformed each gene into the vector form, *V*_*g*_, and the entry of the vector represented the association between the gene and the keyword in the dictionary (eq. 4). As a consequence, the dimension of a vector is the number of keywords contained in the dictionary. For each gene, the sum of values correspondent to keywords in all text vectors was multiplied by *TW*_*i*_ of keywords corresponded to these genes. Disease vectors were transformed in the same approach, *V*_*d*_. A total of 3288 gene vectors and 445 disease vectors were transformed and used to predict gene-disease linkages.

The correlation between gene (*V*_*g*_) and disease (*V*_*d*_) was measured by cosine similarity (eq. 4):

4 $$ \cos <{V}_g,{V}_d>=\frac{V_g\cdot {V}_d}{\mid {V}_g\mid \cdot \mid {V}_d\mid } $$

The precision of prediction was defined as:

$$ P(x)=\frac{\mid \left\{\left(g,d\right):\cos <{V}_g,{V}_d>\kern0.5em \ge x\right\}\cap \left\{\left(g,d\right):\left(g,d\right)\in K\right\}\mid }{\mid \left\{\left(g,d\right):\cos <{V}_g,{V}_d>\kern0.5em \ge x\right\}\mid}\kern0.5em ,\kern0.5em 0\le x\le 1 $$

In which, {(*g*, *d*) : cos  < *V*_*g*_, *V*_*d*_ >   ≥ *x*}represents all gene-disease pairs with angle smaller than *x* and {(*g*, *d*) : (*g*, *d*) ∈ *K*}represents the union set of known gene-disease linkages. As a consequence, *P*(*x*) represents the proportion of known gene-disease linkages among all gene-disease pairs with angle smaller than *x.*

The recall of prediction was defined as:

$$ R(x)=\frac{\mid \left\{\left(g,d\right):\cos <{V}_g,{V}_d>\kern0.5em \ge x\right\}\cap \left\{\left(g,d\right):\left(g,d\right)\in K\right\}\mid }{\mid \left\{\left(g,d\right):\left(g,d\right)\in K\right\}\mid}\kern0.5em ,\kern0.5em 0\le x\le 1 $$

*R*(*x*) represents the proportion of known gene-disease linkages with angle smaller than *x* among all known gene-disease linkages.

## Results

### The overall distribution of cosine similarity value

**Fig. 3:**
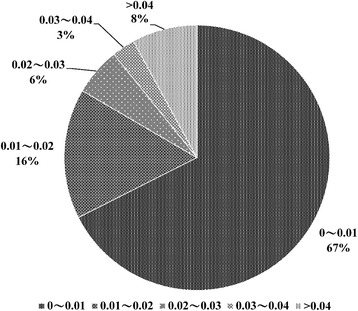
The distribution of cosine similarity of gene-disease pairs. The distribution of cosine similarities of 1,407,672 gene-disease pairs is shown in the pie plot. Gene-disease pairs were binned according their cosine similarities

A total of 1,407,672 values of cosine similarity between 3288 gene vectors and 445 disease vectors were calculated. The distribution of cosine values was shown in the Fig. 3. There were over 67% with cosine values < 0.01 and over 83% that were < 0.02. The distribution of cosine similarities of gene-disease pair showed that, in general, most genes were not associated with diseases. This distribution also demonstrated that for each disease, only a few of genes might be related with it respectively.

### Evaluating the performance of cosine similarity in predicting gene-disease linkage

**Fig. 4:**
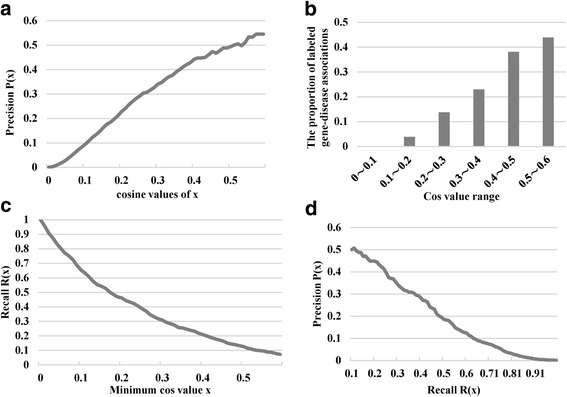
The relationship between precision rate, recall rate, and cosine similarity. **a** The precision rate increases with increasing cosine similarity. **b** The proportion of labeled gene-disease associations among different cosine similarity ranges is shown. **c** The relationship between recall rate and cosine similarity is shown. **d** The tradeoff between precision and recall is shown

First, we investigated the relationship between cosine similarity and precision rate. As results shown in the Fig. 4a, the precision rate increased with increments in cosine similarity. In addition, when cosine similarity was greater than 0.5, the precision remained stable around 0.6. Among the gene-disease pairs with cosine similarity greater than 0.5, over half of them were annotated in the OMIM database. Furthermore, there were only 2 gene-disease pairs with cosine similarity smaller than 0.9 and both of them were also annotated as known linkages. This demonstrated that the predictability of cosine similarity in aspect of the gene-disease linkage. Fig. 4b showed the proportion of labeled gene-disease associations with cosine similarity greater than *x* among different cosine similarity ranges. The proportion of OMIM-annotated gene-disease associations increased with cosine similarity. Figure 4c shows that the recall rate decreases with increasing cosine similarity and it also demonstrated the discriminant power of cosine similarity in predicting gene-disease linkages. Figure 4d shows the tradeoff relationship between precision rate and recall rate.

### The effects of applying weight matrix, PWK, and normalization

**Fig. 5:**
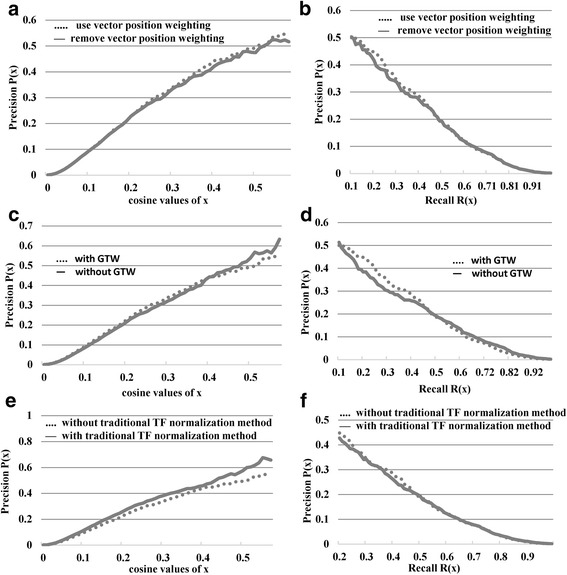
The effects of applying weight matrix in the text vectorization step. The effects of applying weight matrix in the text vectorization step are shown in the relationship between (**a**) precision rate and cosine similarity and (**b**) the precision and recall rates. The solid line represents results obtained without using the weight matrix and the dashed line represents those obtained with the weight matrix. The effects of applying PWK in penalizing the depth of the keyword in the MeSH are shown in the relationship between (**c**) precision rate and cosine similarity and (**d**) the precision and recall rates. The solid line represents results obtained without PWK and the dashed line represents those obtained with PWK. The effects of applying TF normalization are shown in the relationship between (**e**) precision rate and cosine similarity and (**f**) the precision and recall rates. The solid line represents results obtained with TF normalization and the dashed line represents those without TF normalization

The effects of applying the weight matrix in the text vectorization step were shown in Fig. 5a and b. Results showed that the precision rate was marginally improved with the weight matrix when cosine similarity value was greater than 0.3 or recall rate was smaller than 0.4. Because the region with high precision rate or low recall rate is more meaningful in aspect of gene-disease linkage prediction, applying the weight matrix is meaningful in improving the prediction performance.

The effects of applying PWK in penalizing the depth of the keyword in the MeSH were shown in the Figs. 5c and d. Keywords without specificity may introduce more error while not information and, as a consequence, decreased the power and accuracy of prediction. PWK penalized keywords without specificity in terms of disease association and decreased the effects of these keywords. Although results also showed that without PWK penalization the precision was marginally higher in gene-disease pairs with higher cosine similarity, the precision rate with PWK penalization was higher in the low recall rate region, than the precision rate without PWK penalization (Fig. 5d). Nevertheless, these findings show that the PWK penalization does improve the overall performance of gene-disease association prediction in high precision rate and low recall rate regions.

Comparisons of TF normalization methods were shown in the Fig. 5e and f. Although, the precision rate of applying the standardized normalization method was stochastically higher than the precision rate of applying the log-transformation method, it was caused by the standardized normalization method enlarged the effects of text documents containing fewer keywords while decreased the effects of text documents containing more keywords. This may introduce a bias of overweighting short text documents. As a consequence, we concluded that the log-transformation method outperformed standardized normalization method in high precision rate and low recall rate regions (Fig. 5f).

### Comparison with HNEP

**Fig. 6:**
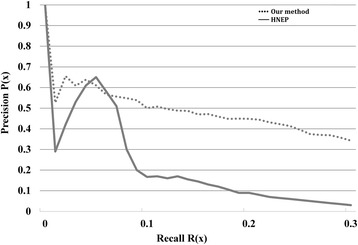
Comparison with the Heterogeneous Network Edge Prediction (HNEP) method. Our method was compared with the HNEP method based on the precision-recall curve. The solid line represents the HNEP method and the dashed line represents our method

We compared our method with HNEP method [30]. HNEP is a method that integrates the graphic model and MLM to predict gene-disease linkages based on logistic regression analysis. We found that the precision rate of our method was significantly higher than the precision rate of HNEP when the recall rate higher than 0.1 and marginally higher when the recall rate lower than 0.1 and (Fig. 6). As a consequence, we concluded that out method outperformed the HNEP method in predicting gene-disease linkages.

## Discussion

In this study, we predicted potential gene-disease linkages using text documents associated with gene names or disease names in the PubMed, MeSH, and OMIM databases. We transformed keywords in the dictionary to vectors to represent genes or diseases, respectively, and then calculated the cosine similarity between gene vectors and disease vectors. Although we took PubMed as the source data, our method could be generalized to other database fields with records described by nature language.

One of the novelty of our method is to consider the specificity of the keyword. Remarkably, our method not only adapts the concept of TF-IDF that bridges genes and diseases through term frequencies in the dictionary but also reweight the keywords according to the MeSH tree. The main reason is to penalize those keywords without specificity meaning such as “family” which may not happen frequently and still have high value in the IDF. PWK will penalize the words without specificity meaning because they are very close to the root of the MeSH tree.

Although the DISEASES study [21] investigated co-occurrence of gene and disease in the text document, it focused on analyzing known gene-disease linkages but did not predict unknown gene-disease pairs. HNEP [30] and CATAPULT [8] both provided prediction results but they did not integrate text documents with their methods. LGscore [32] focused on associations between genes with less consideration about disease, limiting the application LGscore in only some specific diseases. Our prediction method of gene-disease linkage, described in this study, not only utilized information from text documents in PubMed and keywords in MeSH, but also considered the keyword frequency distribution to adjust the weight matrix. As a consequence, our method can be readily adapted to predict more gene-disease linkages, even in the case of diseases that have not been widely studied.

Gene-disease pairs with higher association predicted by our method tended to overlap known gene-disease pairs annotated by OMIM. As a consequence, gene-disease pairs with high cosine similarity, especially those without known annotation, may be valuable for further investigating their association. Furthermore, based on our results, the importance of associated genes could be ranked in one specific disease and this gene rank may do help to disease-associated gene exploration in the disease of interest. Also, a similar protocol for prioritization of diseases when studying the impact of specific genes can be performed using our method.

One potential general application of our method is that not only text documents in PubMed, but also results of other studies, can be integrated into the current graphic model. Such integration may yield a better performance for gene-disease association predictions. In addition, one potential extension of our method is that gene-gene or disease-disease associations could also be inferred using our method.

## Conclusion

In this study, we proposed a MLM of predicting potential gene-disease linkages by mining gene or disease related text documents and evaluated the performance of prediction results by comparing the data with those of another method, HNEP. Results of our prediction method quantified potential gene-disease linkages. The novelty of our method is based on the combination of text mining and the graphic model. To our knowledge, there is currently no graphic model involving the kind of dataset described herein. As a consequence, our method may provide new avenues for exploring gene-disease linkages, improving prediction performance, and combining widely-used current graphic models.

## Abbreviations

HNEP: Heterogeneous Network Edge Prediction
IDF: Inverse document frequency
MLM: Machine learning method
TF-IDF: Term frequency–inverse document frequency
